# Reconstitution of the core of the malaria parasite glideosome with recombinant *Plasmodium* class XIV myosin A and *Plasmodium* actin

**DOI:** 10.1074/jbc.M117.813972

**Published:** 2017-10-04

**Authors:** Carol S. Bookwalter, Chwen L. Tay, Rama McCrorie, Michael J. Previs, Hailong Lu, Elena B. Krementsova, Patricia M. Fagnant, Jake Baum, Kathleen M. Trybus

**Affiliations:** From the ‡ Department of Molecular Physiology and Biophysics, University of Vermont, Burlington, Vermont 05405 and; the § Department of Life Sciences, Imperial College London, South Kensington, London SW7 2AZ, United Kingdom

**Keywords:** actin, chaperone, malaria, myosin, parasite, Plasmodium falciparum, myosin XIV, glideosome, malaria, myosin light chain, MTIP, UCS family chaperone

## Abstract

Motility of the apicomplexan malaria parasite *Plasmodium falciparum* is enabled by a multiprotein glideosome complex, whose core is the class XIV myosin motor, PfMyoA, and a divergent *Plasmodium* actin (PfAct1). Parasite motility is necessary for host-cell invasion and virulence, but studying its molecular basis has been hampered by unavailability of sufficient amounts of PfMyoA. Here, we expressed milligram quantities of functional full-length PfMyoA with the baculovirus/*Sf*9 cell expression system, which required a UCS (UNC-45/CRO1/She4p) family myosin chaperone from *Plasmodium* spp. In addition to the known light chain myosin tail interacting protein (MTIP), we identified an essential light chain (PfELC) that co-purified with PfMyoA isolated from parasite lysates. The speed at which PfMyoA moved actin was fastest with both light chains bound, consistent with the light chain–binding domain acting as a lever arm to amplify nucleotide-dependent motions in the motor domain. Surprisingly, PfELC binding to the heavy chain required that MTIP also be bound to the heavy chain, unlike MTIP that bound the heavy chain independently of PfELC. Neither the presence of calcium nor deletion of the MTIP N-terminal extension changed the speed of actin movement. Of note, PfMyoA moved filaments formed from *Sf*9 cell–expressed PfAct1 at the same speed as skeletal muscle actin. Duty ratio estimates suggested that as few as nine motors can power actin movement at maximal speed, a feature that may be necessitated by the dynamic nature of *Plasmodium* actin filaments in the parasite. In summary, we have reconstituted the essential core of the glideosome, enabling drug targeting of both of its core components to inhibit parasite invasion.

## Introduction

Malaria is a blood-borne disease that causes nearly half a million deaths per year (1) and is caused by apicomplexan parasites of the genus *Plasmodium*. The force required for the parasite to invade vertebrate host hepatocytes or erythrocytes is powered by a multiprotein assembly called the glideosome, the core of which is the class XIV myosin PfMyoA, which interacts with a divergent and dynamic *Plasmodium* actin isoform (PfAct1) (2). Class XIV myosins are monomeric with a short heavy chain. The motor domain, which binds actin and MgATP, is followed by a light chain–binding region of ∼50 amino acids but no further tail. Typically, myosins have well-defined IQ motifs (consensus sequence IQ*XXX*RG*XXX*R) following the motor domain, which are known to bind EF-hand proteins such as myosin light chains and calmodulin. The neck of PfMyoA potentially contains two degenerate IQ motifs, but at present there is only one light chain known to bind to this region of the PfMyoA heavy chain, called myosin tail interacting protein (MTIP).^4^ The last 15 amino acids of the heavy chain are sufficient to bind MTIP (3), and MTIP has been crystallized in several conformations bound to this heavy-chain peptide (4, 5). The nomenclature for MTIP derived from the idea that it bound to the tail of the molecule, a region which typically acts as a targeting and/or a dimerizing domain. One model of glideosome organization proposes that PfMyoA is anchored via the ∼60 amino acid N-terminal extension of MTIP to integral membrane proteins (glideosome-associated proteins, or GAPs) located in a double-membraned flattened complex called the inner-membrane complex, which lies ∼25 nm below the plasma membrane (2). In that capacity, part of MTIP can functionally act like a tail.

Despite the central importance of the *Plasmodium* actomyosin complex to cellular invasion of red blood cells and development of malaria, progress at the molecular level has been hampered by the inability to express milligram quantities of PfMyoA that are needed to investigate its biochemical and biophysical properties and to identify small molecule inhibitors to potentially disrupt invasion. Moreover, although *Plasmodium* spp. actin has been expressed recombinantly (6), its ability to interact with PfMyoA has never been shown *in vitro*, and it has been suggested that *Plasmodium* actin is incompletely folded when expressed in heterologous expression systems (7).

Here, we show that *Sf*9 cell expression of functional class XIV *Plasmodium* MyoA requires a UCS family (UNC-45/CRO1/She4p) myosin chaperone derived from *Plasmodium* spp., similar to our previous observation that the distantly related apicomplexan *Toxoplasma gondii* MyoA required a myosin chaperone from *T. gondii* for proper folding (8). UCS family myosin co-chaperones have three domains: an N-terminal tetratricopeptide repeat that binds to the general chaperone HSP-90; a central domain of unknown function; and a C-terminal UCS domain that binds the myosin motor domain (9). We also identified a novel essential-type light chain (PfELC) that is unexpectedly divergent from the previously identified essential light chains from *T. gondii* (10, 11) and that required the presence of MTIP to bind to the heavy chain. Only when both MTIP and the newly identified PfELC are bound does PfMyoA move actin at fast speeds (∼3.8 μm/s). Expressed PfAct1 formed filaments that were moved by PfMyoA at speeds indistinguishable from those obtained using skeletal muscle actin, despite the divergence of the two actins. Our ability to express functional PfMyoA and PfACT1 in a heterologous expression system allows the motor and its track to be characterized structurally and functionally, and it allows the first *in vitro* reconstitution of the core of the malaria parasite glideosome.

## Results

### Expression of PfMyoA in Sf9 cells requires co-expression with a Plasmodium UCS family chaperone

We expressed two *Plasmodium falciparum* class XIV myosin heavy-chain constructs using the baculovirus/*Sf*9 insect cell-expression system. One construct was the motor domain (PfMD), which contains the actin-binding and active site. It consisted of heavy-chain amino acids Met-1–Lys-768, followed by a FLAG tag to facilitate purification by affinity chromatography. The second construct was the full-length PfMyoA that also included the light chain–binding domain, followed by a biotin tag for attachment to neutravidin-coated surfaces for *in vitro* motility assays and a C-terminal FLAG tag for affinity purification. The full-length construct was expressed in combination with the known light chain MTIP, which interacts with the C-terminal 15 amino acids of the PfMyoA heavy chain (3).

**Figure 1.**
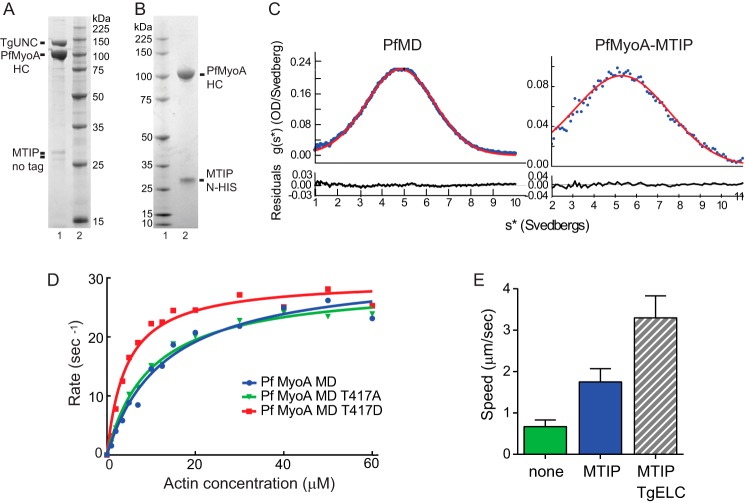
**Characterization of expressed PfMyoA and PfMD.** *A, lane 1,* full-length PfMyoA heavy chain was co-expressed with MTIP and TgUNC, the myosin co-chaperone from *T. gondii* (8), and purified on a FLAG-affinity column. Note that the chaperone, TgUNC, co-purifies with the myosin, indicating that some of the heavy chain is misfolded. *HC,* heavy chain. *Lane 2,* molecular mass markers. 12% SDS gel. *B, lane 1,* molecular mass markers. *Lane 2,* full-length PfMyoA heavy chain was co-expressed with MTIP and the *Plasmodium* myosin co-chaperone, PUNC. In contrast to *A*, the chaperone did not co-purify with the myosin, indicating a properly folded protein. 4–12% SDS gel. *C,* truncated heavy-chain motor domain (*MD*) construct, PfMD sedimented at 5.0 ± 0.01 S. Full-length PfMyoA heavy chain co-expressed with MTIP sedimented at 5.7 ± 0.02 S. A single symmetrical peak is indicative of protein homogeneity. Solvent conditions: 10 mm HEPES, pH 7.5, 0.15 m NaCl (0.1 m NaCl for PfMD), 1 mm DTT, 20 °C. *OD*, optical density. *D,* plots of ATPase rate *versus* actin concentration for wild-type PfMyoA MD (*blue-filled circles*), MD (T417A) (*green inverted triangles*), and MD (T417D) (*red-filled squares*). Data were fit to the Michaelis-Menten equation to yield *V*_max_ values of 31.7 ± 1.7 s^−1^ for WT, 29.1 ± 0.8 s^−1^ for T417A, and 29.7 ± 0.9 s^−1^ for T417D. *K_m_* values were 13 ± 1.8 μm for WT, 9.8 ± 2.9 μm for T417A, and, 4.4 ± 0.5 μm for T417D. *Errors* are S.E. of the fit. Conditions: 10 mm imidazole, 5 mm NaCl, 1 mm MgCl_2_, 2 mm MgATP, 1 mm NaN_3_, 1 mm DTT at 30 °C. *E,* full-length PfMyoA heavy chain co-expressed with no light chain (*green*) moved actin at an average ± S.D. speed of 0.67 ± 0.16 (*n* = 1008 filaments, two experiments and two protein preparations). PfMyoA with bound MTIP (*blue*) moved actin at 1.75 ± 0.32 μm/s (*n* = 3242 filaments, four experiments and two protein preparations). PfMyoA with both MTIP and TgELC bound (*gray stripes*) moved actin at 3.3 ± 0.53 μm/s (*n* = 1691 filaments, two experiments and one protein preparation). The difference in speed between all pairs was significant (*t* test, *p* < 0.001). Conditions: 50 mm KCl, 25 mm imidazole, pH 7.5, 1 mm EGTA, 4 mm MgCl_2_, 10 mm DTT, 2 mm MgATP, 30 °C.

When *Sf*9 cells were infected with virus encoding the PfMyoA heavy chain and MTIP, the heavy chain was expressed but was not soluble following cell lysis. This result suggested that the endogenous *Sf*9 cell chaperones were not capable of properly folding the PfMyoA heavy chain. Co-expression of FLAG-tagged full-length PfMyoA heavy chain, untagged MTIP, and the UCS myosin co-chaperone from the apicomplexan *T. gondii* (TgUNC) (8) produced soluble protein, but a considerable amount of TgUNC remained bound to PfMyoA following purification on a FLAG affinity resin (Fig. 1*A*). Chaperones only bind to unfolded proteins and are released once the protein is folded; thus, TgUNC could not fulfill the same role in folding that it did for its native class XIV myosin, TgMyoA (8). We speculated that the homologous chaperone from *Plasmodium* spp. was required.

A BLAST search using TgUNC as input identified a putative tetratricopeptide repeat family protein, which was ∼90 amino acids longer than most other UNC proteins, which could be the *P. falciparum* myosin co-chaperone (accession number PF3D7_1420200, nomenclature according to http://PlasmoDB.org or GenBank^TM^ XP_001348369.1).^5^ Our first attempts at expression of the putative chaperone by itself, with native *Plasmodium* codons, resulted in no detectable quantity of protein being expressed in *Sf*9 cells. Expressing proteins from *Plasmodium* spp. in heterologous cells is challenging due to its extremely AT-rich genome, which results in large differences from *Sf*9 cell codon preference (12). Another issue is that regions of the putative chaperone contained atypically long stretches of asparagine residues. To circumvent the latter problem, potential chaperone protein sequences from eight different *Plasmodium* spp. were aligned, and conserved areas were identified. A chimeric protein was designed by deleting or changing the *P. falciparum* coding sequence to that of *Plasmodium knowlesi* in regions where the *P. falciparum* sequence was significantly different from the consensus sequence of the other seven species (supplemental Fig. 1). To address preferred codon usage, the chimeric chaperone was synthesized using *Sf*9 cell preferred codons. We refer hereafter to the putative chimeric chaperone as PUNC (*Plasmodium* spp. UNC).

Co-expression of PUNC with FLAG-tagged full-length PfMyoA heavy chain and N-His-tagged MTIP produced soluble PfMyoA, which was purified free of the PUNC chaperone, consistent with a fully folded protein that releases the chaperone (Fig. 1*B*). By analytical ultracentrifugation, PfMyoA–MTIP sedimented as a single symmetrical boundary with a sedimentation coefficient of 5.7 ± 0.02 S (Fig. 1*C*). This result verifies the homogeneity of the expressed protein in solution and is strong evidence for proper folding of the myosin by PUNC. Soluble protein was also obtained when the shorter PfMD construct was co-expressed with PUNC. Affinity-purified PfMD also showed a single symmetrical peak, with a sedimentation coefficient of 5.0 ± 0.01 S (Fig. 1*C*). The smaller sedimentation coefficient is consistent with the lower molecular weight of PfMD.

An unusual feature of MTIP was that it migrated as a single band on SDS gels only when it was tagged at the N terminus, whether expressed in *Sf*9 cells or in *Escherichia coli*. Expression with untagged or C-terminally tagged MTIP resulted in multiple gel bands (Fig. 1, *A* and *B*). MTIP immunoprecipitated from *Plasmodium* lysates has also been shown to run as multiple bands on SDS gels, and it was suggested that this may be the result of various phosphorylation states (13). Mass spectrometry data of N-HIS-MTIP expressed in *Sf*9 cells showed that it was multiply phosphorylated (Ser-51, -55, -58, and -61), yet it migrated as a single band once N-terminally tagged. One possibility is that the His_6_ tag suppresses proteolytic cleavage at the N-terminal region.

### A phosphomimic at the TEDS site enhances affinity for actin

With few exceptions, analysis of the heavy chain of many myosin classes showed that the base of a surface loop (“cardiomyopathy loop”) that interacts with actin has either an acidic residue (Asp/Glu) or a residue capable of being phosphorylated (Thr/Ser), which introduces a negative charge. Because of the residues involved it is called the TEDS rule site (14). PfMyoA has a threonine (Thr-417) in the TEDS site, implying that phosphorylation of this residue may regulate actomyosin affinity. We engineered a phospho-null T417A mutation and a phosphomimic T417D mutation at this site in the PfMD construct. Actin-activated ATPase assays on the two MD mutants were compared with values obtained with wild-type PfMD. ATPase activity as a function of actin concentration (Fig. 1*D*) showed that the *V*_max_ was similar for all three constructs (31.7 ± 1.7 s^−1^ for WT, 29.1 ± 0.8 s^−1^ for T417A, and 29.7 ± 0.9 s^−1^ for T417D; ±S.E. of the fit). The actin concentration at half-maximal activity (*K_m_*), which reflects the affinity of the motor for actin, depended on the charge at the TEDS site. Both the WT PfMD and the T417A mutant had similar *K_m_* values (13 ± 1.8 μm for WT and 9.8 ± 2.9 μm for T417A; ± S.E. of the fit), consistent with mass spectrometry data showing that Thr-417 of PfMyoA was not phosphorylated during expression in *Sf*9 cells. In contrast, the *K_m_* value of the phosphomimic T417D was over 2-fold lower (4.4 ± 0.5 μm), indicating a higher affinity for actin. ATPase assays are more capable of revealing differences in affinity for actin than motility assays, which are performed in the presence of methylcellulose, which prevents actin filaments from diffusing away from the surface. Although phosphorylation of this site has not yet been detected in proteomic studies of *P. falciparum* parasites, these data raise the intriguing possibility that transient phosphorylation at this site could be a mechanism to enhance motor engagement when actin filaments are sparse.

### In vitro motility of PfMyoA

The function of expressed full-length PfMyoA was assessed by an *in vitro* motility assay, where surface-bound motors propel rhodamine–phalloidin-labeled actin in solution. PfMyoA was specifically attached by its C-terminal biotin tag to a neutravidin-coated coverslip to ensure that all motor domains were available to interact with skeletal actin. When the full-length PfMyoA heavy chain was expressed without light chains, it moved actin at a speed of 0.67 ± 0.16 μm/s (average ± S.D., *n* = 1008 filaments, data from two experiments and two protein preparations) (Fig. 1*E*, *green bar*). When the heavy chain was co-expressed with MTIP, the speed increased to 1.75 ± 0.32 μm/s (*n* = 3242 filaments, four experiments and two protein preparations) (Fig. 1*E*, *blue bar*). Speeds were analyzed using a semi-automated filament tracking program (see “Experimental procedures”) and fit to a Gaussian curve. Use of this program permitted evaluation of a large number of filaments without selection bias by the user. The faster *in vitro* motility speed with MTIP bound is consistent with the idea that speed is proportional to the effective length of the lever arm, which depends on the number of light chains bound to a given myosin (15).

We speculated that PfMyoA may bind a second light chain, based on the observation that the class XIV MyoA from *T. gondii* binds an essential-type light chain (ELC) (8, 10, 11) in addition to MLC1, the homolog of MTIP. For proof of principle, PfMyoA heavy chain, MTIP, and TgELC were co-expressed. TgELC bound to the PfMyoA heavy chain and increased *in vitro* motility speeds to 3.3 ± 0.53 μm/s (*n* = 1691 filaments, two experiments and one protein preparation). The same increase in speed was observed when TgELC was added to surface-adhered PfMyoA–MTIP just prior to visualization of motion, indicating that the heavy chain co-expressed with MTIP remains competent to rebind an essential-type light chain. This result strongly implies that PfMyoA binds an essential-type light chain in addition to MTIP. Given that the binding site for MTIP is known to be the last 15 amino acids of the heavy chain, the ELC must bind to heavy-chain sequences C-terminal to the converter region of the motor domain and N-terminal to the MTIP-binding site, as is true of essential light chains of other myosins.

### Database search for the P. falciparum essential light chain

To identify potential ELCs in *P. falciparum*, a BLAST search was performed using TgELC as input. The highest BLAST score was for *P. falciparum* calmodulin (PfCaM, PF3D7_1434200/GenBank^TM^ XP_001348497, 45% identity with TgELC). Calmodulin is the “light chain” for a number of classes of unconventional vertebrate myosins, *e.g.* class V and class VI. Co-expression of PfMyoA heavy chain and MTIP with PfCaM in *Sf*9 cells showed no PfCaM bound to the purified myosin. Addition of PfCaM to the final motility buffer showed no enhancement in speed, either in the presence or absence of calcium, in contrast to the doubling of speed observed upon addition of TgELC (Table 1). Other candidate light chains whose functional impact was assayed are documented in Table 1. All candidates tested failed to meet our functional criteria of a *bona fide* ELC, namely that speed should increase in an *in vitro* motility assay, and the putative ELC should co-purify with the PfMyoA heavy chain. We concluded that the endogenous essential light chain was divergent from TgELC and could only be identified from pulldowns from *Plasmodium* parasite lysates.

**Table 1:** ***In vitro* motility speeds of PfMyoA-MTIP to which various putative light chains were added** PfMyoA heavy chain with a C-terminal biotin tag, co-expressed with MTIP, was bound to a neutravidin-coated coverslip. The indicated essential light chain was added to the final *in vitro* motility buffer. TgELC1 is a known essential light chain that binds to TgMyoA from *T. gondii* (8, 10, 11). *n*, number of actin filaments analyzed. The last entry, PF3D7_1017500, is the PfELC identified here.

PlasmoDB or ToxoDB ID of added light chain	GenBank^TM^ accession no.	% identity with TgELC	Annotation	Calcium	Speed
					μ*m/s*
No added light chain				−	1.8 ± 0.3 (*n* = 3242)
TGME49_269442	XP_002365635.1	100	TgELC1	−	3.3 ± 0.5 (*n* = 1691)
PF3D7_1434200	XP_001348497	45	*P. falciparum* calmodulin	−	1.7 ± 0.3 (*n* = 1122)
				+	1.3 ± 0.2 (*n* = 1612)
PF3D7_1418300	XP_001348354.1	26	PF14_0181 Putative calmodulin	−	No motility
PF3D7_0627200	XP_966255.2	21	PFF1320c Putative myosin light chain	−	1.1 ± 0.2 (*n* = 1249)
				+	0.6 ± 0.2 (*n* = 1466)
PF3D7_0107000	SBT75303.1	35	PfPC1Putative centrin-1	−	1.5 ± 0.2 (*n* = 1265)
PF3D7_1017500	XP_001347455.1	20	PfELC Conserved *Plasmodium* protein	−	3.8 ± 0.5 (*n* = 2732)
				+	3.8 ± 0.6 (*n* = 1776)

### Identification of the P. falciparum essential light chain

**Figure 2.**
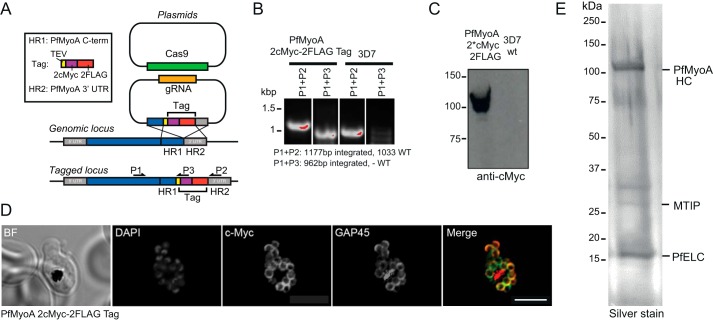
**Tagging and localization of the PfELC.** *A,* schematic illustrating the strategy used to tag PfMyoA with a C-terminal 2cMyc-FLAG tag using CRISPR/CAS9. Two plasmids were transfected into *P. falciparum* 3D7 ring stage parasites, one plasmid carrying the guide DNA sequence (AGCTCATATAAGAAAAAAAA) and the desired tag flanked by a fragment of the PfMyoA C terminus and a fragment of the PfMyoA 3′-UTR (5′- and 3′-homology regions), and a second plasmid containing the cassette for Cas9 expression. Double crossover homologous recombination results in the insertion of the tag sequence between the C-terminal end of PfMyoA and the PfMyoA 3′-UTR. Primers used for detection of the integration of the tag sequence into the genomic loci are shown. *B,* PCR analysis of PfMyoA-2cMyc-2FLAG-tag transgenic parasites. Primers able to differentiate between WT sequence and integration of the tag sequence into genomic loci were used to confirm the insertion of the desired tag into the PfMyoA locus. *Empty lanes* between samples have been removed for clarity, although they derive from the same gel. *C,* Western blot of PfMyoA-2cMyc-2FLAG-tag transgenic parasites probed with anti-c-Myc *versus* control untagged 3D7 line. *D,* immunofluorescence assay using anti-c-Myc (*green*), anti-GAP45 (*red*), and DAPI (*blue*) in PfMyoA-2cMyc-2FLAG-tag transgenic parasites shows the successful expression of the C-terminal tag and confirmed that the tagged PfMyoA still localized to its expected location in schizonts (at the periphery). *BF,* bright field. *Scale bar,* 5 μm. *E,* silver stain on 12% SDS-PAGE with bands identified as the PfMyoA complex along with the newly identified PfELC.

To assess whether an additional light chain could be identified directly from the parasite, the PfMyoA heavy chain was tagged in *P. falciparum* using CRISPR/CAS9 technology (16) to introduce a C-terminal dual FLAG tag and a double c-Myc epitope tag (Fig. 2*A*). After parasite cloning, insertion of the 2cMyc–2FLAG-tag sequence into the 3′-end of the PfMyoA gene locus was confirmed via PCR analysis (Fig. 2*B*). Expression of the C-terminal tag in *P. falciparum* schizont stage parasites was verified via Western blotting (Fig. 2*C*) and an immunofluorescence assay using antibodies against the c-Myc epitope (Fig. 2*D*). Large-scale cultures were grown and used to purify PfMyoA (Fig. 2*E*). Comparing proteins precipitated by FLAG *versus* control untagged parasite lysates, a 15-kDa light chain-like protein was identified by mass spectrometry as PF3D7_1017500 (GenBank^TM^ accession number XP_001347455.1) (see supplemental Dataset S1). Of note, no other obvious light chain or calmodulin-like proteins were identified that were specifically pulled down in FLAG *versus* control untagged preparations.

**Figure 3.**
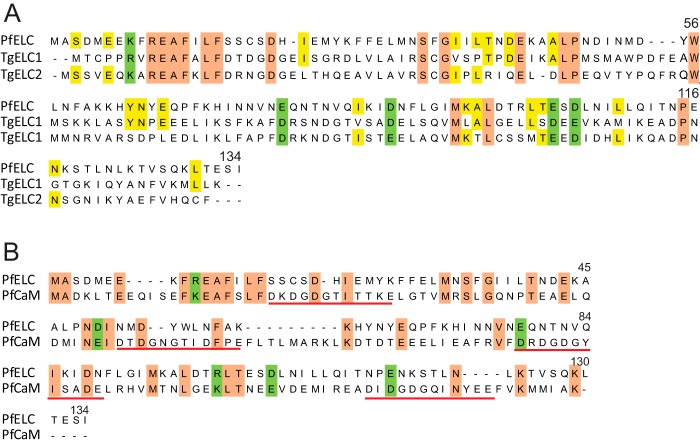
**Sequence comparisons with the novel PfELC.** *A,* sequence comparison of the PfELC identified here (Plasmo DB PF3D7_1017500, GenBank^TM^ XP_001347455.1) with essential light chain isoforms from *T. gondii*, TgELC1 (Toxo DB TGME49_269442, GenBank^TM^ XP_002365635.1) and TgELC2 (Toxo DB TGME49_305050, GenBank^TM^ XP_018636125.1) (10, 11). Identity in all three proteins is shown in *salmon* and identity in PfELC and one of the TgELC isoforms in *yellow*. Common charged residues (Asp/Glu or Arg/Lys) present in all three light chains are indicated in *green*. PfELC is 20% identical to TgELC1 and 21% identical to TgELC2. *B,* sequence comparison of the PfELC identified here and Pf calmodulin (PfCaM) (Plasmo DB PfCaM PF3D7_1434200, GenBank^TM^ XP_001348497). Identity is shown in *salmon* and common charged residues (Asp/Glu or Arg/Lys) in *green*. The four calcium-binding sites in PfCaM are indicated by the *horizontal red lines*. Calcium binding requires a Asp/Glu at position 12 of the motif, and thus PfELC does not appear to contain a functional calcium-binding site.

The newly identified PfELC is only 20% identical to TgELC1 (Toxo DB TGME49_269442, GenBank^TM^ XP_002365635.1) and 21% identical with TgELC2 (Toxo DB TGME49_305050, GenBank^TM^ XP_018636125.1), validating why it was not picked up as one of the top hits from the database search (Fig. 3*A*). Moreover, a sequence comparison with PfCaM shows that the newly identified PfELC is unlikely to have any functional calcium-binding sites, as it lacks the requisite acidic residue (Asp/Glu) at position 12 of the calcium-binding motif (Fig. 3*B*).

### PfELC binding enhances motility speed

**Figure 4.**
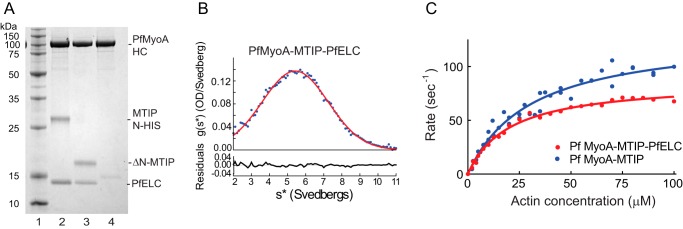
**PfELC requires the presence of MTIP to bind to the heavy chain and actin-activated ATPase activity of PfMyoA.** *A, lane 1,* molecular mass markers. *Lanes 2* and *3,* PfMyoA heavy chain and PUNC co-expressed with PfELC, and either MTIP or ΔN-MTIP binds both classes of light chains. *Lane 4,* when the PfMyoA heavy chain and PUNC are co-expressed only with PfELC, the light chain does not bind with high enough affinity to co-purify with the heavy chain. 12% SDS gel. *B,* full-length PfMyoA heavy chain co-expressed with MTIP and the newly identified PfELC sedimented at 5.7 ± 0.01 S. A single symmetrical peak is indicative of protein homogeneity. Solvent conditions: 10 mm HEPES, pH 7.5, 0.15 m NaCl, 1 mm DTT, 20 °C. *OD*, optical density. *C,* ATPase rate as a function of actin concentration for full-length PfMyoA with only MTIP bound (*blue*) or with both PfELC and MTIP bound (*red*). Data were fit to the Michaelis-Menten equation to yield *V*_max_ = 130 ± 6 s^−1^ and *K_m_* = 31 ± 3 μm for PfMyoA–MTIP (four experiments and two protein preparations), and *V*_max_ = 87 ± 2 s^−1^ and *K_m_* = 20 ± 1 μm for PfMyoA with both light chains (two experiments and one protein preparation). *Errors* are S.E. of the fit. Conditions: 10 mm imidazole, 5 mm NaCl, 1 mm MgCl_2_, 2 mm MgATP, 1 mm NaN_3_, 1 mm DTT at 30 °C.

**Figure 5.**
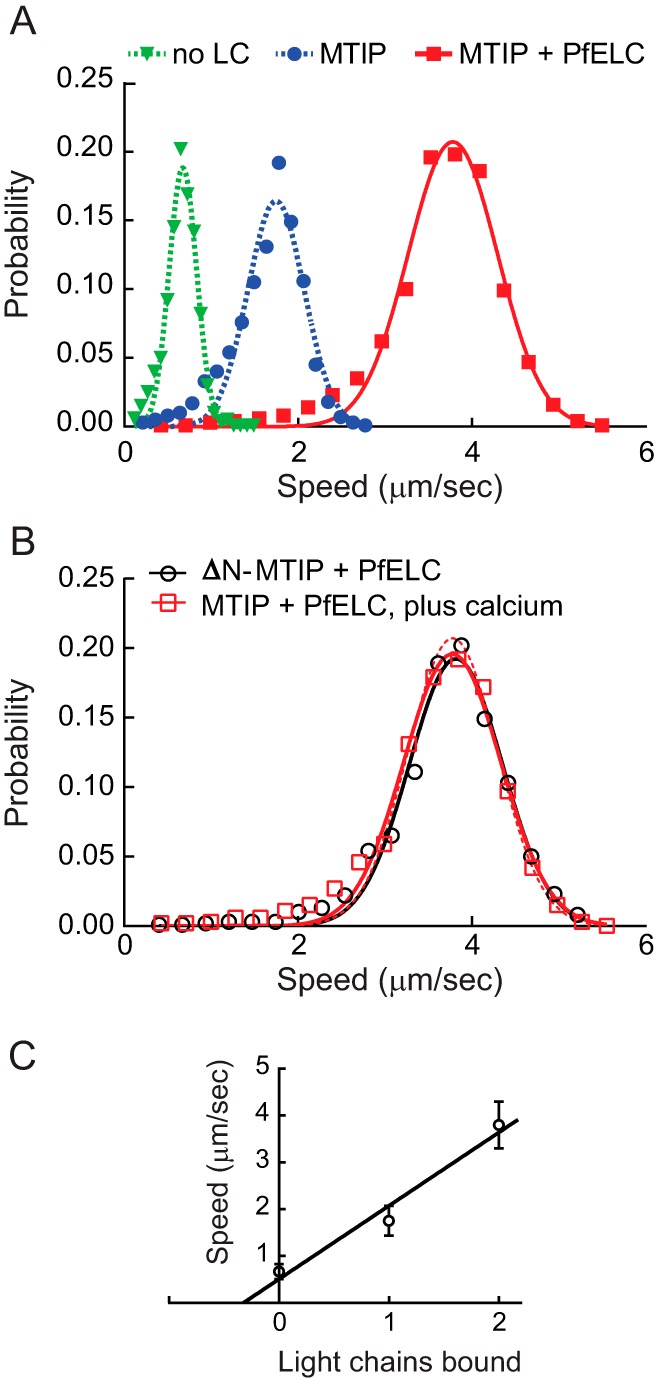
***In vitro* motility of PfMyoA with PfELC and MTIP.** *A,* speed distributions of full-length PfMyoA with no light chain bound (*green*), 0.67 ± 0.16 μm/s (*n* = 1008 filaments, two experiments); with MTIP bound (*blue*), 1.75 ± 0.32 μm/s (*n* = 3242 filaments, four experiments); or with both MTIP and PfELC bound (*red*), 3.78 ± 0.52 μm/s (*n* = 2732 filaments, five experiments). *Errors* are mean ± S.D. *B, in vitro* motility speeds of PfMyoA with both bound light chains in the presence of 100 μm calcium (*open red squares*) was 3.78 ± 0.56 μm/s (*n* = 1776 filaments, two experiments, *open red squares*). Speed at which PfMyoA containing an N-terminal truncation of MTIP (ΔN-MTIP) and PfELC moved actin at 3.82 ± 0.54 μm/s (*n* = 1433 filaments, two experiments, *open black circles*). Data fit from *A* with PfELC and MTIP is repeated in *B* with *red dashed lines*. Neither speed is significantly different from the control (*t* test, *p* > 0.05). Conditions for *A* and *B*: 50 mm KCl, 25 mm imidazole, pH 7.5, 1 mm EGTA (except 100 μm CaCl_2_ where indicated in *B*), 4 mm MgCl_2_, 10 mm DTT, 2 mm MgATP, 30 °C. *C,* speed correlates linearly with number of light chains bound to the heavy chain.

To assess the ability of PfELC to bind the PfMyoA heavy chain, PfELC was co-expressed in *Sf*9 cells with the PUNC chaperone, PfMyoA heavy chain, and MTIP. The purified myosin showed an intact heavy chain and two bound light chains by SDS gels (Fig. 4, *lane 2*) and sedimented as a single homogeneous 5.7 ± 0.01 S peak by sedimentation velocity (Fig. 4*B*). This was the same sedimentation coefficient as PfMyoA–MTIP, suggesting that the increase in mass due to the bound PfELC is balanced by an increase in asymmetry due to stabilization of the asymmetric light chain–binding domain by two light chains. Importantly, the *in vitro* motility speed of PfMyoA with both MTIP and PfELC bound was 3.78 ± 0.52 μm/s (*n* = 2732 filaments combined from five experiments and two protein preparations), faster than when either no light chain or only MTIP was bound (Fig. 5*A*). There was a linear relationship between speed and number of light chains bound (Fig. 5*C*), similar to the linear relationship between unitary displacement and lever arm length previously observed with vertebrate class II myosins (17). The *x*-intercept of −0.16 suggests that the fulcrum of the lever arm extends into the base of the motor domain as observed with more conventional class II myosins (17). These *in vitro* data confirm that the newly identified PfELC from the parasite functions as a *bona fide* light chain for PfMyoA.

To examine whether the long N-terminal extension present in MTIP affects the speed at which the motor moves actin, we cloned a truncated version of MTIP that lacked the first 60 residues (ΔN-MTIP) for co-expression with the heavy chain. Light-chain extensions in skeletal muscle myosin have been shown to interact with actin and slow the speed of actin movement (18). Removal of the N-terminal extension of MTIP, however, showed the same speed of actin movement (3.82 ± 0.54 μm/s, *n* = 1433 filaments, two experiments and one protein preparation) (Fig. 5*B*, *open black circles*), as with the full-length MTIP. This result is consistent with the proposed role of the N-terminal extension of MTIP as a binding partner of the GAP proteins, rather than engaging in an interaction with actin.

### Binding of PfELC requires MTIP

Co-expression experiments showed that for PfELC to bind to the heavy chain, either MTIP or ΔN-MTIP must also be co-expressed. Fig. 4*A*, *lane 4,* shows that no PfELC bound to the heavy chain when it was the only light chain co-expressed with the heavy chain. This observation is in stark contrast to the ability of MTIP to bind tightly and co-purify with the heavy chain in the absence of PfELC (Fig. 1*B*) and to yield a homogeneous protein that retains the ability to recruit an ELC following purification.

### Calcium does not change speed

Addition of 100 μm calcium caused no change in speed (3.78 μm/s ± 0.56, *n* = 1776 filaments, two experiments and one protein preparation) relative to the speed in buffer containing EGTA (Fig. 5*B*, *open red squares*). Four mm MgCl_2_ was present in the motility buffer both in the absence or presence of calcium, which could in principle compete for potential divalent cation-binding sites. Sequence analysis, however, implies that PfELC does not have a functional calcium-binding site (Fig. 3*B*).

### Total ATPase cycle time

The actin-activated ATPase activity of full-length PfMyoA with MTIP alone, or with both MTIP and PfELC bound, was measured so that the total cycle time could be determined. Data were fit to the Michaelis-Menten equation. For PfMyoA–MTIP, the maximal velocity (*V*_max_) was 130 ± 6 s^−1^, and the actin concentration at half-maximal velocity (*K_m_*) was 31 ± 3 μm (±S.E. of the fit) (Fig. 4*C*). For PfMyoA with both MTIP and PfELC bound, *V*_max_ = 87 ± 2 s^−1^ and *K_m_* = 20 ± 1 μm (±S.E. of the fit). Total cycle times are thus 7.7 ms for PfMyo-MTIP and 11.5 ms with both light chains bound.

### Comparison of motility with PfAct1 versus skeletal muscle actin

**Figure 6.**
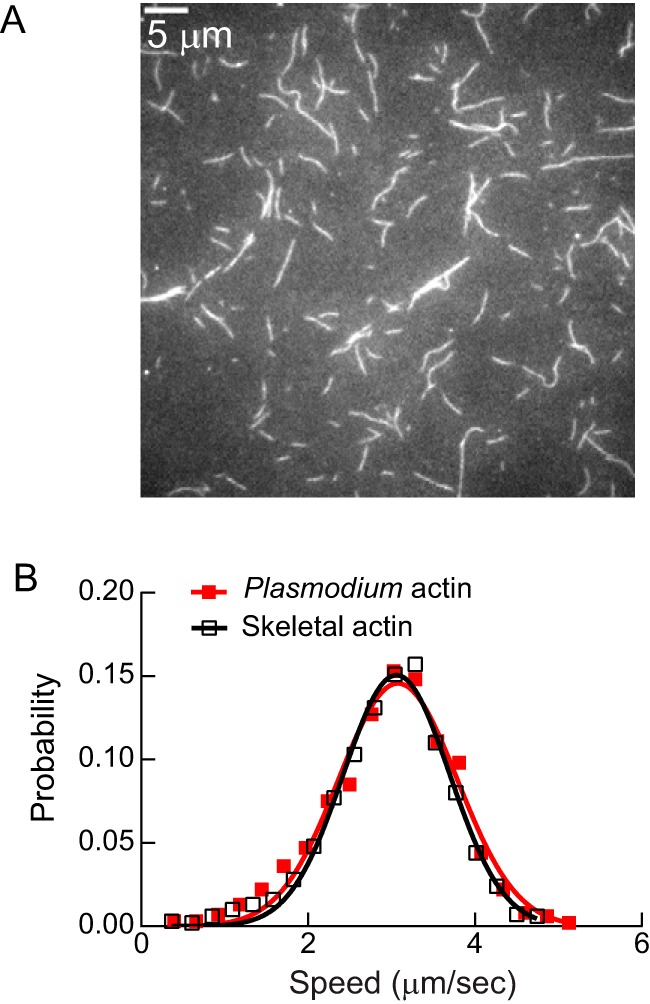
**Motility speed with skeletal actin *versus Plasmodium* actin.** *A,* filaments formed from expressed *Plasmodium* actin, stabilized with jasplakinolide, and visualized with actin–chromobody Emerald using TIRF microscopy. *Bar,* 5 μm. *B,* speed at which PfMyoA moved skeletal actin (3.08 ± 0.71 μm/s, *n* = 1372 filaments, two experiments, *open black squares*) or *Plasmodium* actin (3.06 ± 0.64 μm/s, *n* = 1128 filaments, two experiments and two actin preparations, *red-filled squares*) were not significantly different (*t* test, *p* > 0.05). Both filaments were stabilized with jasplakinolide and visualized with actin–chromobody Emerald using TIRF microscopy. See supplemental Movies 1 and 2. Conditions: 25 mm imidazole, pH 7.5, 100 mm KCl, 1 mm EGTA, 4 mm MgCl_2_, 10 mm DTT, 2 mm MgATP, 30 °C.

PfAct1 was expressed in *Sf*9 cells so that the interaction of PfMyoA with its native actin could be investigated. All prior motility experiments and ATPase assays described here were done with skeletal actin, which is 82% identical with PfAct1. Rhodamine–phalloidin, used to visualize skeletal actin filaments, did not bind to *Plasmodium* actin filaments nor did SiR-actin, a fluorescent jasplakinolide derivative, and thus an alternative method was necessary. We chose to visualize the filaments with actin–chromobody Emerald, which has been used to visualize F-actin dynamics in *T. gondii* (19). Both skeletal actin filaments and *Plasmodium* actin filaments were stabilized with jasplakinolide to ensure a direct comparison between the two types of filaments. Fig. 6*A* shows a field of jasplakinolide-stabilized *Plasmodium* actin filaments visualized by TIRF microscopy with actin–chromobody Emerald. The presence of the actin–chromobody did not interfere with the ability of PfMyoA (MTIP and PfELC) to move actin. Moreover, the speed at which PfMyoA moved *Plasmodium* actin (3.06 ± 0.64 μm/s, *n* = 1128 filaments, two experiments and two actin preparations) was indistinguishable from that obtained with skeletal actin under identical conditions (3.08 ± 0.71 μm/s, *n* = 1372 filaments) (Fig. 6*B*). Supplemental Movies S1 and S2 show the excellent quality of the motility with both actins. Although this result does not preclude finding a difference between the two actins under some conditions, such as under load, the similar speeds at which PfMyoA moves both its cognate actin and a heterologous actin was surprisingly unaffected under unloaded conditions.

## Discussion

Reconstitution of the malaria parasite glideosome requires expression of its key components, the class XIV myosin motor PfMyoA and the actin track with which it interacts, PfAct1. Here, we expressed milligram quantities of functional PfMyoA in *Sf*9 cells by co-expressing the heavy chain with a *Plasmodium* spp. myosin co-chaperone of the UCS family (PUNC). Expressed PfMyoA moves actin in an *in vitro* motility assay at the fastest speed (∼3.8 μm/s) when two light chains are bound: the previously identified MTIP (3, 4) and PfELC, a novel essential-type light chain identified here from mass spectrometry analysis of proteins that co-purified with tagged PfMyoA heavy chain from *Plasmodium* parasites. The domain structure of PfMyoA is thus similar to conventional myosins, with a motor domain that binds actin and ATP and a light chain–binding domain that acts as a lever arm. We also show that filaments formed from PfAct1 expressed in *Sf*9 cells can be moved by PfMyoA at speeds indistinguishable from those seen with skeletal actin, under unloaded conditions.

### Chaperone specificity

Many myosins can utilize the chaperones present in *Sf*9 cells to properly fold, consistent with chaperone–client interactions being multifunctional (20). Among the exceptions are the class XIV MyoA from *T. gondii,* which required TgUNC (8), and here we show that *P. falciparum* class XIV MyoA requires its own genus-specific myosin co-chaperone, PUNC. It was recently shown that depletion of TgUNC from *T. gondii* parasites destabilizes all 11 myosins from five classes, implying that TgUNC plays a role in folding all myosins (21). This finding implies that successful expression of any of these myosins (TgMyoA through TgMyoI) in a heterologous system may require co-expression with TgUNC. It remains to be determined whether PUNC plays a similar role in folding all six myosins (PfMyoA to PfMyoF) in *Plasmodium* spp. parasites.

Like the class XIV myosins, striated muscle myosins (cardiac, skeletal) also failed to utilize endogenous *Sf*9 cell chaperones for folding. This led to the development of the myocyte cell line C2C12 for striated muscle myosin expression, which by definition has all the factors needed to fold muscle myosins (22). Once all the chaperones and associated proteins necessary for striated muscle myosin folding are identified, it is likely that expression of functional striated myosin will be possible in *Sf*9 cells. Another example is myosin 15, native to inner ear hair cells, which unexpectedly required co-expression with the muscle-specific chaperone UNC45b and heat-shock protein 90 to properly fold in *Sf*9 cells (23).

### Identification of the PfMyoA essential light chain

The last 15 amino acids of the PfMyoA heavy chain are sufficient to bind MTIP (3), leaving enough residues between the C terminus of the motor domain and the beginning of the MTIP-binding site to bind another light chain. Here, we identified a novel essential light chain, PfELC, which bound to a tagged PfMyoA heavy chain that was precipitated from large-scale parasite cultures. It was unexpectedly divergent from the previously identified essential light chain isoforms in *T. gondii* (TgELC1 and TgELC2) that bind mutually exclusively to the TgMyoA heavy chain (10, 11). This finding is a lesson that native light chains are best identified by association with the native heavy chain *in vivo*, rather than database searches. Secondary and tertiary protein prediction using the RaptorX web portal (http://raptorx.uchicago.edu/)^5^ predicted that PfELC has seven helices (58% helical), connected by loops, typical of a light chain that could bind to and stabilize a single α-helical myosin heavy chain.

In support of its association with the glideosome, PfELC was also previously found in a proteomic study of proteins greatly reduced in abundance following selective inhibition of *N*-myristoylation, which leads to failure to assemble the inner-membrane complex incorporating the major glideosome-associated proteins (24). In addition, the newly identified PfELC bound to the PfMyoA heavy chain when co-expressed in *Sf*9 cells, and it doubled the *in vitro* motility speed compared with PfMyoA containing MTIP alone. PfELC thus appears to be a *bona fide* light chain of the PfMyoA-dependent glideosome.

An unusual feature of PfELC is that it required the presence of MTIP during co-expression in *Sf*9 cells to bind to the heavy chain. This result suggests that MTIP may enhance the affinity of PfELC for the heavy chain, either through a direct interaction between the two light chains or by favoring a conformation of the heavy chain that has enhanced affinity for PfELC. Protein preparations expressed in the absence of MTIP also contain small amounts of cleavage at the C terminus of the heavy chain, indicating a general destabilization of the light chain–binding region. An interesting implication of this finding is that if MTIP binding is disrupted, the whole lever arm becomes destabilized and the ability to move actin will be seriously compromised.

### Calcium and the essential light chain

The first isoform of the essential light chain from *T. gondii* (TgELC1) was identified from an analysis of proteins that bound more tightly to TgMyoA in the presence of calcium (10). The residues in TgELC1 predicted to chelate calcium were Asp-15, Asp-17, and Asp-19 (11). In PfELC, only one of these three residues (Asp-21 in PfELC) is conserved (Fig. 3*A*); the other two residues are Ser. Alignment of known calcium-binding sites in PfCaM with potential motifs in PfELC also suggests that PfELC is not capable of binding calcium (Fig. 3*B*). Functionally, we observed no difference in the speed of actin movement with PfMyoA in the presence or absence of 100 μm calcium, with 4 mm MgCl_2_ present. Either PfELC does not bind a divalent cation or under our buffer conditions the site is occupied by magnesium, which is present at a 40-fold higher concentration than calcium. The free calcium concentration in the *Plasmodium* cytoplasm has been measured to be only 350 nm (25). Consistent with our results, an earlier study in which a small amount of PfMyoA was isolated from parasites and analyzed by *in vitro* motility showed no difference in speed between *p*Ca <8 to *p*Ca 4.1 (13). Remarkably, their speed of movement in the motility assay was 3.5 μm/s, very similar to the 3.8 μm/s measured here. In the former study, it is likely that the essential light chain was bound to the heavy chain but not identified on the SDS gels used due to its small size (∼15 kDa).

### Duty ratio estimates

The duty ratio of PfMyoA can be estimated from the time spent strongly attached to actin relative to the total ATPase cycle time. Assuming a unitary step-size of ∼5.5 nm (*d*_uni_) for PfMyoA with both PfELC and MTIP bound (based on 6 nm for single-headed class II myosins with two bound light chains (26) or 5.3 nm for TgMyoA (27)), and a measured speed of 3.8 μm/s (*v*) in the motility assay, yields a strongly attached time (*t*_on_) of ∼1.4 ms (*t*_on_ = *d*_uni_/*v*). The maximal actin-activated ATPase activity of 87 s^−1^ gives a total cycle time of 11.5 ms. The estimated duty ratio for PfMyoA is thus ∼12%. A similar calculation for PfMyoA with only MTIP bound (7.7-ms total cycle time, 1.6-ms strongly attached time, assumed step-size of ∼2.8 nm, which is half that with both light chains) predicts a duty ratio of ∼20%. Both values are greater than the 3–4% duty ratios of conventional class II myosins that work in large filamentous ensembles, but less than the minimum 50% duty ratio that is required of myosins that transport cargo as a single motor (28). A duty ratio of ∼12% implies that even small ensembles of motors (∼9) will move actin at maximal speed. The architecture of the glideosome and the labile and potentially short actin filaments in the parasite may necessitate a higher duty ratio motor.

### Interaction of PfMyoA with Plasmodium versus skeletal actin

*Plasmodium* actin 1 (PfAct1) is markedly divergent from skeletal actin, having only ∼80% sequence identity with mammalian actins, a large difference considering the near identity of most actins (6). Nevertheless, when we compared the speed at which PfMyoA moved skeletal actin *versus* expressed *Plasmodium* actin in an unloaded *in vitro* motility assay, we saw no difference. This result does not rule out finding a difference between the two actins using other assays or conditions in the future. A challenge in performing these experiments was finding a way to visualize the *Plasmodium* actin filaments, which did not bind either rhodamine–phalloidin or SiR–actin, a fluorescent jasplakinolide derivative. Actin–chromobody Emerald, which was recently used to visualize F-actin dynamics in *T. gondii* (19), proved to be a good way to observe motility of *Plasmodium* actin *in vitro*.

We noted that the speed at which *Plasmodium* actin was moved by PfMyoA appeared to not be very sensitive to ionic strength (50–150 mm KCl). This result implies that the interface on PfMyoA that interacts with actin may involve fewer ionic interactions than with these other myosins. Loop 2 of PfMyoA, a positively charged surface loop that is involved in the initial electrostatic interaction with actin, has fewer charged residues and is at least 15 amino acids shorter than either smooth muscle myosin or myosin V.

It has been suggested that *Plasmodium* actin is incompletely folded in heterologous systems (7) and that this could explain its aberrant polymerization behavior. Our data do not rule out this possibility, but they do suggest that *Plasmodium* actin expressed in *Sf*9 cells folds properly to the extent that it provides a binding surface for PfMyoA, which can then be moved in a manner similar to skeletal muscle actin isolated from tissue.

### Unique features of PfMyoA compared with TgMyoA

Despite both being class XIV myosins from apicomplexan parasites, PfMyoA and TgMyoA show some unique features. First, each myosin requires its own native chaperone for proper folding in *Sf*9 cells. TgUNC did not fully substitute for PUNC in folding the PfMyoA motor domain. Second, PfMyoA conforms to the TEDS site rule in having a phosphorylatable Thr-417 residue at the base of a key actin-binding loop in the myosin heavy chain (14). We showed that a negative charge at this residue enhanced actin affinity, implying that phosphorylation has the potential to regulate actomyosin activity *in vivo*. High-resolution cryo-EM reconstructions of an actin–class I myosin complex showed that a negatively charged TEDS site residue interacted with a neighboring Lys residue to stabilize the actin-binding loop (29). Adjacent to Thr-417 in the PfMyoA heavy chain is Lys-418, which could play a similar role. TgMyoA, in contrast, is an exception in that it contains Gln at the TEDS site, which is neither negatively charged nor phosphorylatable. Finally, the newly identified PfELC and TgELC are divergent, with only 20–21% sequence identity. PfELC appears to have no functional calcium-binding site, whereas TgELC can bind calcium and may regulate some features of this motor (10, 11).

## Conclusions

The speeds we measured under unloaded conditions (∼3.8 μm/s) exceed that needed for an ∼1-μm merozoite to invade a red blood cell in less than 20 s (0.05 μm/s) (30). The force–velocity curve of myosin shows that speed decreases under load, and thus our measured speeds are compatible with the biology of cellular invasion. Our results solidify the idea that the domain structure of class XIV myosins consists of a motor domain followed by two light chains that bind to the C-terminal region of the heavy chain and act as a lever arm. In the absence of a true tail, the N-terminal region of MTIP likely plays that role by anchoring the motor into the inner-membrane complex via integral GAP proteins. The ability to express PfMyoA in milligram quantities allows needed structural and functional studies to be performed with the motor that powers invasion of host cells to cause malaria, a disease that is a major global health issue. PfMyoA and PfAct1 are also novel druggable targets for inhibition of cellular invasion.

## Experimental procedures

### Expression constructs

The full-length PfMyoA heavy chain (PlasmoDB ID PF3D7_1342600/GenBank^TM^ accession number XM_001350111.1) was obtained by PCR using gBlocks® gene fragments **(**Integrated DNA Technologies) with *Sf*9 cell preferred codons as template. A 13-amino acid linker (NVSPATVQPAFGS) was added to the C terminus of the PfMyoA heavy chain to separate it from an 88-amino acid segment of the *E. coli* biotin carboxyl carrier protein (31, 32) that gets biotinylated during expression in *Sf*9 cells, followed by a C-terminal FLAG tag. The PCR product was cloned into the baculovirus transfer vector pAcSG2 (BD Biosciences) to make recombinant virus.

A shorter PfMyoA motor domain construct that lacked the light chain–binding region was truncated at residue Lys-768, followed by a Gly-Ser linker and a C-terminal FLAG tag. This heavy-chain fragment was cloned into baculovirus transfer vector pAcSG2 (BD Biosciences). Two other constructs, in which the TEDS site motif Thr (Thr-417) was mutated to either Ala (phospho-null) or Asp (phosphomimic), were also cloned.

The *PfMTIP* gene (PF3D7_1246400/GenBank^TM^ accession number XM_001350813.1) was PCR-amplified from gBlocks® gene fragments using *Sf*9-preferred codons and cloned with an N-terminal His_6_ tag in both pAcSG2 for *Sf*9 cell expression and pET19 (Novagen) for bacterial expression. In the ΔN-MTIP version, the 60-amino acid N-terminal extension of both MTIP-pAcSG2 and MTIP-pET19 was removed by site-directed mutagenesis. The N-His tag was retained, and MTIP begins with Ser-61.

Four potential essential light chain candidates were cloned using *Sf*9-preferred codons into a bacterial expression vector, and additionally three of these were cloned in a baculovirus vector. PF3D7_1434200 (GenBank^TM^ accession number XM_001348461.1) had an N-terminal His_6_ tag in both pFastBac and pET19. PF3D7_1418300 (GenBank^TM^ accession number XM_001348318.1) was C-terminally His_6_-tagged in both pAcSG2 and pET3. PF3D7_0627200 (GenBank^TM^ accession number XM_961162.2) had an N-terminal His tag in pFastBac and no tag in pET3. PF3D7_0107000 (GenBank^TM^ accession number SBT75303) was only cloned for bacterial expression using pET19 with an N-terminal His_6_ tag. TgELC used as a control was described previously (8).

The *PfELC* gene (PF3D7_1017500/GenBank^TM^ accession number XM_001347419.1) identified here was cloned using *Sf*9 preferred codons using gBlocks® gene fragments. The resulting PCR product was cloned into the bacterial expression vector pET19 with an N-terminal His_6_ tag. For expression in *Sf*9 cells, it was cloned without a tag into pFastBac (Thermo Fisher Scientific) for recombinant virus production.

The *Plasmodium* UCS family chaperone, herein called PUNC, is a chimeric clone made using the coding sequence of *P. falciparum* (PF3D7_1420200/GenBank^TM^ accession number XM_001348333.1) but substituting sequences from *P. knowlesi* (PKNH_1337800/GenBank^TM^ accession number XM_002260772) in regions that *P. falciparum* did not show consensus among other *Plasmodium* species (supplemental Fig. 1). A PCR product was made using gBlocks® gene fragments with *Sf*9-preferred codons. The resulting chaperone, with a Myc tag at the C terminus, was cloned into the baculovirus transfer vector pAcSG2 (BD Biosciences) for recombinant virus production. TgUNC used as a control was described previously (8).

*Plasmodium* actin 1 (PF3D7_1246200/GenBank^TM^ accession number XM_001350811.1) followed by a linker and human thymosin-β4 was synthesized using gBlocks® gene fragments with *Sf*9-preferred codons and cloned into pAcUW51 for expression in *Sf*9 cells as described previously (33).

### Myosin expression and purification

The full-length PfMyoA heavy chain or the MD was co-expressed with the co-chaperone PUNC in *Sf*9 cells. As indicated, either a His_6_-tagged MTIP alone or both MTIP and an essential-type light chain were co-expressed. The cells were grown for 72 h in medium supplemented with 0.2 mg/ml biotin, harvested, and lysed by sonication in 10 mm imidazole, pH 7.4, 0.2 m NaCl, 1 mm EGTA, 5 mm MgCl_2_, 7% (w/v) sucrose, 2 mm DTT, 0.5 mm 4-(2-aminoethyl)benzenesulfonyl fluoride, 5 μg/ml leupeptin, and 2.5 mm MgATP. An additional 2.5 mm MgATP was added prior to clarifying at 200,000 × *g* for 40 min. The supernatant was purified by FLAG-affinity chromatography (Sigma). The column was washed with 10 mm imidazole, pH 7.4, 0.2 m NaCl, and 1 mm EGTA. The myosin was eluted from the column in the same buffer containing 0.1 mg/ml FLAG peptide. The fractions of interest were combined, concentrated with an Amicon centrifugal filter device (Millipore), and dialyzed against 10 mm imidazole, pH 7.4, 0.2 m NaCl, 1 mm EGTA, 55% (v/v) glycerol, 1 mm DTT, and 1 μg/ml leupeptin for storage at −20 °C.

### Light chain expression and purification

Bacterially expressed MTIP and PfELC His-tagged light chains were expressed in BLR(DE3)-competent cells and grown in LB broth. Cultures were grown overnight at 27 °C following induction with 0.7 mm isopropyl β-d-thiogalactopyranoside, after which the pellet was harvested and frozen. Pellets were lysed by sonication in 10 mm sodium phosphate, pH 7.4, 0.3 m NaCl, 0.5% (v/v) glycerol, 7% (w/v) sucrose, 7 mm β-mercaptoethanol, 0.5 mm 4-(2 aminoethyl)benzenesulfonyl fluoride, and 5 μg/ml leupeptin. The cell lysate was clarified at 26,000 × *g* for 30 min, after which the supernatant was boiled for 10 min in a double boiler, clarified at 26,000 × *g* for 30 min, and the supernatant was loaded on a His-Select nickel-affinity column (Sigma). The resin was washed in buffer A (10 mm sodium phosphate, pH 7.4, and 0.3 m NaCl) before eluting with buffer A containing 200 mm imidazole. The protein was concentrated and dialyzed overnight against 10 mm imidazole, pH 7.4, 150 mm NaCl, 1 mm EGTA, 1 mm MgCl_2_, 1 mm DTT, and 55% (v/v) glycerol and stored at −20 °C.

### Plasmodium actin expression, purification, and filament formation

Expression and purification of *Plasmodium* actin 1 was essentially as described in Ref. 33. Infected *Sf*9 cells (2 × 10^9^) were harvested 3 days after infection and lysed by sonication in 50 ml of 10 mm HEPES, pH 8.0, 0.25 mm CaCl_2_, 0.3 m NaCl, 7 mm β-mercaptoethanol, 0.25 mm Na_2_ATP, clarified, and immediately bound to a nickel-affinity column. Non-specifically bound protein was washed off with 2 column volumes of 10 mm imidazole, pH 8.0, 10 mm HEPES, pH 8.0, 0.25 mm CaCl_2_, 0.3 m NaCl, 7 mm β-mercaptoethanol, 0.25 mm Na_2_ATP, 1 μg/ml leupeptin. Actin was eluted with wash buffer containing 200 mm imidazole. The thymosin and His tag were cleaved off with a 1:20 weight ratio of chymotrypsin/actin. Actin was separated from the thymosin–His tag using a Mono Q 5/50 GL column (GE Healthcare) with a gradient of 0–0.3 m NaCl in 5 mm Tris, pH 8.26, at 4 °C, 0.2 mm CaCl_2_, 0.1 mm NaN_3_, 0.5 mm DTT, 0.2 mm Na_2_ATP, and 1 μg/ml leupeptin, followed by a step to 0.5 m NaCl. Peak fractions were pooled, concentrated, and dialyzed against 5 mm Tris, pH 8.26, at 4 °C, 0.2 mm CaCl_2_, 0.1 mm NaN_3_, 0.5 mm DTT, 0.2 mm Na_2_ATP, and 1 μg/ml leupeptin. Before use, 3 mg of *Plasmodium* G-actin were applied to a Superdex 10/300 GL column (GE Healthcare) equilibrated with 5 mm Tris, pH 8.2, at 4 °C, 0.2 mm CaCl_2_, 0.5 mm DTT, and 0.2 mm NaATP. Fractions eluting at the position of monomeric actin were pooled, and the concentration was determined using the Bio-Rad protein assay. Filaments were formed immediately by first exchanging calcium for magnesium by addition of 0.2 mm EGTA and 0.05 mm MgCl_2_ for 5 min at 4 °C. Polymerization was then initiated by addition of 10 mm imidazole, pH 7.4, 50 mm KCl, 4 mm MgCl_2_, 1 mm EGTA, and a 1.1-fold molar excess of jasplakinolide (Invitrogen) relative to G-actin. The mixture was incubated for 1 h at 37 °C.

### Actin-chromobody expression and purification

Actin-chromobody (ChromoTek Inc., Hauppauge, NY) with a C-terminal EmeraldFP followed by a C-terminal His_6_ tag for purification was cloned into a pET22b vector (Novagen) (plasmid was a gift from Markus Meissner, University of Glasgow, and Aoife Heaslip, University of Connecticut). Protein was expressed in BL-21 (DE3) cells that were induced with 0.05 mm IPTG and grown overnight at 20 °C in LB broth. The cells were lysed by sonication in 10 mm sodium phosphate, pH 7.4, 0.3 m NaCl, 0.5% glycerol, 7% sucrose, 0.5 mm DTT, 0.5 mm 4-(2-aminoethyl)benzenesulfonyl fluoride, 0.5 mm l-1-chloro-3-[4-tosylamido]-7-amino-2-heptanone HCl, and 5 μg/ml leupeptin. The lysate was clarified at 200,000 × *g* for 30 min, and the supernatant was applied to His-Select nickel-affinity gel (Sigma). The resin was washed with 10 mm sodium phosphate, 10 mm imidazole, pH 7.4, 0.3 m NaCl, 0.5 mm DTT, and 1 μg/ml leupeptin and then eluted from the column with 10 mm sodium phosphate, 200 mm imidazole, pH 7.4, 0.3 m NaCl, 0.5 mm DTT, and 1 μg/ml leupeptin. The protein was dialyzed against 10 mm imidazole, pH 7.4, 0.3 m NaCl, 1 mm EGTA, 1 mm MgCl_2_, 1 mm DTT, 10 μg/ml leupeptin, 50% glycerol and stored at −20 °C.

### Sedimentation velocity

Sedimentation velocity runs were performed at 20 °C in an Optima XL-I analytical ultracentrifuge (Beckman Coulter) using an An60Ti rotor at 40,000 rpm. The solvent was 10 mm HEPES, pH 7.5, 0.1 or 0.15 m NaCl, and 1 mm DTT. The sedimentation coefficient was determined by curve fitting to one species using the d*c*/d*t* program (34).

### In vitro motility

A nitrocellulose-coated flow cell was prepared by adding 0.5 mg/ml biotinylated bovine serum albumin (BSA) in buffer A (150 mm KCl, 25 mm imidazole, pH 7.5, 1 mm EGTA, 4 mm MgCl_2_, and 10 mm DTT) for 1 min followed by three rinses with 0.5 mg/ml BSA in buffer A. The flow cell was then infused with 50 μg/ml neutravidin (Thermo Fisher Scientific) in buffer A for 1 min followed by three rinses with buffer A. Before use, PfMyoA was mixed with a 3-fold molar excess of actin and 5 mm MgATP and spun for 20 min at 350,000 × *g* to remove myosin that was unable to dissociate from actin in the presence of MgATP. The protein concentration of the supernatant was determined using the Bio-Rad Protein assay. PfMyoA (0.5 μm), which had a biotin tag at the C terminus of the heavy chain, was infused into the neutravidin-coated flow cell and incubated for 1 min and then rinsed three times with buffer B (50 mm KCl, 25 mm imidazole, pH 7.5, 1 mm EGTA, 4 mm MgCl_2_, and 10 mm DTT). The chamber was then washed two times with 1 μm unlabeled F-actin in buffer B, which was first sheared by vortexing, to block any remaining MgATP-insensitive motors. The flow cell was then washed three times with buffer B containing 2 mm MgATP, followed by three washes with buffer B. Rhodamine–phalloidin-labeled actin was then introduced for 30 s, followed by one rinse with buffer B, and one rinse with buffer C. Buffer C is buffer B plus 0.5% (w/v) methylcellulose, 25 μg/ml PfMTIP, and 25 μg/ml PfELC (or whatever essential light chain was being tested), and oxygen scavengers (50 μg/ml catalase (Sigma), 125 μg/ml glucose oxidase (Sigma), and 3 mg/ml glucose). Finally, buffer C containing 2 mm MgATP was flowed twice through the flow cell. Assays were performed at 30 °C. When assays were performed in the presence of calcium, the light chains were dialyzed against buffer containing 0.1 mm calcium and no EGTA, and 1 mm EGTA in the motility buffers was replaced with 0.1 mm calcium.

Actin movement was observed at 30 °C using an inverted microscope (Zeiss Axiovert 10) equipped with epifluorescence, a Rolera MGi Plus digital camera, and dedicated computer with the Nikon NIS Elements software package. Data were analyzed using a semi-automated filament tracking program described previously (35). The speeds of >1000 filaments were determined. Speeds were fit to a Gaussian curve.

### In vitro motility comparing Plasmodium and skeletal actin by TIRF microscopy

Experiments in which the *in vitro* motility of *Plasmodium versus* skeletal actin filaments was compared differed from the above protocol in the following ways. (i) There was no wash with unlabeled actin. (ii) Both skeletal and *Plasmodium* actin filaments were stabilized with jasplakinolide. (iii) 300 nm actin–chromobody Emerald (ChromoTek Inc., Hauppauge, NY) (plasmid was a gift from Markus Meissner, University of Glasgow, and Aoife Heaslip, University of Connecticut) was included in the final motility buffer for visualization of filaments. (iv) The KCl concentration in the final assay buffer was 100 mm instead of 50 mm. The use of the actin–chromobody necessitated the use of TIRF microscopy. The assay was carried out on a Nikon ECLIPSE Ti microscope, run by the Nikon NIS Elements software package, and equipped with through-objective type TIRF and a temperature control unit that enclosed the flow cell. The samples were excited with the TIRF field of a 488-nm laser line, and emission was observed with a 525/50 filter. The fluorescence image was observed with a ×100 objective and recorded on an Andor EMCCD camera (Andor Technology, South Windsor, CT) at 5 frames/s for 24 s with automatic focus correction. The final resolution is 0.1066 μm/pixel.

### SDS gels

Proteins were separated on either a 12% or a 4–12% gradient BisTris NuPAGE gel (Invitrogen) and run in MOPS or MES buffer according to the NuPAGE technical guide.

### Actin-activated ATPase activity

The actin-activated ATPase activity of PfMyoA was determined at 30 °C in 10 mm imidazole, 5 mm NaCl, 1 mm MgCl_2_, 1 mm NaN_3_, 1 mm DTT as a function of skeletal actin concentration. A low salt concentration was needed to keep the *K_m_* values as low as possible so that *V*_max_ could be achieved. PfMyoA–MTIP concentration was 35 nm, PfMyoA with both light chains was 31.2 nm, and PfMD was 63 nm. The assay was initiated by the addition of 2 mm MgATP and stopped with SDS at four time points every minute for 4 min for full-length PfMyoA constructs or every 1.5 min for PfMD. Inorganic phosphate was determined colorimetrically as described previously (36). Data were fit to the Michaelis-Menten equation.

### Mass spectrometry of Sf9 cell–expressed PfMyoA

The phosphorylation status of MTIP and the TEDS sites on the PfMyoA heavy chain was determined by liquid chromatography-tandem mass spectrometry (LC-MS/MS). The PfMyoA heavy chain 104 kDa) and MTIP (24.6 kDa) bands were separated on SDS-polyacrylamide gels, stained with Coomassie, and excised from the gel. The bands were destained with 50% acetonitrile (ACN), dehydrated with 100% ACN, and dried in a speed vacuum device. The dried bands were rehydrated with 10 mm dithiothreitol (DTT) in 50 mm ammonium bicarbonate (AB) and incubated (55 °C, 45 min). The DTT was removed, and 55 mm iodoacetamide in 50 mm AB was added. The tubes were incubated in the dark (20 °C, 30 min). The iodoacetamide was removed, and the gel slices were rinsed for 15 min with 50% ACN. Rinsing was repeated three times, and the samples were dried in a speed vacuum. A 2-μg aliquot of trypsin (Promega) in 50 mm AB was added to each tube. The tubes were incubated at 4 °C for 1 h and then at 37 °C overnight. An aliquot of 7% formic acid in 50 mm AB was added to each tube. The peptides were serially extracted with 3 aliquots of 50 mm AB, with 15-min incubations between extractions. The resultant peptides were dried in a speed vacuum device and then reconstituted in 0.05% trifluoroacetic acid. The peptides were separated on an Acquity UPLC HSS T3 column (100 Å, 1.8 μm, 1 × 150 mm) (Waters) attached to a Dionex UltiMate 3000 HPLC (Dionex). The HPLC effluent was directly injected into a Q Exactive Hybrid Quadrupole-Orbitrap mass spectrometer through an electrospray ionization source (Thermo Fisher Scientific). Data were collected in data-dependent MS/MS mode with the top five most abundant ions being selected for fragmentation. Peptides were identified from the resultant MS/MS spectra using SEQUEST run via Proteome Discoverer 2.1 software (Thermo Fisher Scientific). These searches were performed against a custom database that reflected the cloned genes (PfMyoA heavy chain and MTIP) plus tags. Peptide oxidation was accounted for by addition of 15.99 and 31.99 Da to each methionine; carbamidomethylation was accounted for by addition of 57.02 Da to each cysteine, and phosphorylation was accounted for by adding 79.97 Da to each serine, threonine, or tyrosine residue. All identifications were manually confirmed by inspection of the MS/MS fragmentation spectra.

Quantitation of phosphorylation ion currents, measured for each identified peptide, were extracted from the MS spectra using PinPoint software (Thermo Fisher Scientific). The degree of phosphorylation was estimated in each sample from the loss in abundance of non-phosphorylated peptides using a mass-balance approach. This loss was determined from the ratio of the non-phosphorylated peptides in each sample to those in samples that had been treated with phosphatase (37, 38).

### Native tagging of PfMyoA

A gene fragment coding for a TEV protease cleavage site followed by the c-Myc epitope tag (2×) and a dual FLAG tag, flanked by a fragment of the C-terminal end of PfMyoA and a fragment of the PfMyoA 3′-UTR, was synthesized by GeneART (Thermo Fisher Scientific) and inserted into the pL6-eGFP CRISPR plasmid (Fig. 2) (16). The pL6-eGFP plasmid was linearized for cloning using the SacII/AflII restriction sites. The guide DNA sequence (AGCTCATATAAGAAAAAAAA) was inserted into the same plasmid using the BtgZI adaptor site (16), producing the completed pL7-PfMyoA-2cMyc-2FLAG-tag plasmid. All cloning steps was performed using Gibson Assembly (39). *P. falciparum* 3D7 strain parasites were maintained in RPMI 1640-based media supplemented with O^+^ human erythrocytes at 4% hematocrit and 0.5% AlbuMAX II (Life Technologies, Inc.), according to established methods (40), with genetic transformation performed as described previously (41). Briefly, ring stage parasites at 8–10% parasitemia were transfected with 60 μg of the pL7-PfMyoA-2cMyc-2FLAG plasmid, along with 60 μg of the pUF1-Cas9 plasmid, which expresses the Cas9 endonuclease (16). Positive drug selection was performed 1 day post-transfection using 2.5 nm WR99210 and 1.5 μm DSM1 and maintained until stable parasite growth was achieved. The transgenic parasites were then cloned by limiting dilution at concentrations of 0.25 parasites/well, 0.5 parasites/well, and 1 parasite/well. Insertion of the tag sequence was determined by PCR analysis of parasite genomic DNA, using primers specific for integration of the tag sequence at the PfMyoA gene locus (Fig. 2). Clones with the integrated tag sequence were then maintained in media without drug for further downstream analysis.

### Large-scale FLAG-tagged immunoprecipitation of PfMyoA-2cMyc-2FLAG culture

3D7 parasites containing FLAG-tagged PfMyoA were maintained in standard cultures and synchronized at ring stage to 8% parasitemia by sorbitol treatment (42). This was further scaled up to 3 liters of culture and harvested at mature schizont stage by lysing cultures with 0.1% saponin/PBS followed by washes with cold PBS to separate lysed erythrocytes from parasites. The parasite pellet was then immediately processed to purify the native PfMyoA complex. Fresh parasite pellet was resuspended in lysis buffer (10 mm imidazole, 300 mm NaCl, 1 mm EGTA, 5 mm MgCl_2_, 1% v/v Triton X-100, 2 mm ATP, 2 mm TCEP) and lysed using a syringe homogenizer on ice. Lysate was centrifuged at 70,000 rpm for 45 min at 4 °C. Anti-FLAG M2 resin (A4596, Sigma) was prepared based on product information and was further washed with lysis buffer. Supernatant of parasite lysate was then mixed with prepared anti-FLAG resin and incubated at 4 °C for 1–2 h. The resin was further washed with wash buffer (10 mm imidazole, 300 mm NaCl, 1 mm EGTA, 5 mm MgCl_2_, 2 mm TCEP) to remove Triton X-100, and protein was eluted using elution buffer (10 mm imidazole, 300 mm NaCl, 1 mm EGTA, 5% glycerol, 2 mm TCEP, 3× FLAG peptide) (F4799, Sigma). Samples were eluted from beads by boiling in Laemmli sample buffer, and the eluates were separated by SDS-PAGE until the dye front had moved 1 cm into separating gel. The gel lane was excised and subjected to in-gel tryptic digestion using a DigestPro automated digestion unit (Intavis Ltd.). Resulting peptides were fractionated using an Ultimate 3000 nano-HPLC system in line with an LTQ-Orbitrap Velos mass spectrometer (Thermo Fisher Scientific). In brief, peptides in 1% (v/v) formic acid were injected onto an Acclaim PepMap C18 nano-trap column (Thermo Fisher Scientific). After washing with 0.5% (v/v) acetonitrile, 0.1% (v/v) formic acid, peptides were resolved on a 250-mm × 75-μm Acclaim PepMap C18 reverse-phase analytical column (Thermo Fisher Scientific) over a 150-min organic gradient, using 7 gradient segments (1–6% solvent B over 1 min, 6–15% B over 58 min, 15–32% B over 58 min, 32–40% B over 5 min, 40–90% B over 1 min, held at 90% B for 6 min, and then reduced to 1% B over 1 min) with a flow rate of 300 nl min^−1^. Solvent A was 0.1% formic acid and solvent B was aqueous 80% acetonitrile in 0.1% formic acid. Peptides were ionized by nano-electrospray ionization at 2.1 kV using a stainless steel emitter with an internal diameter of 30 μm (Thermo Fisher Scientific) and a capillary temperature of 250 °C. Tandem mass spectra were acquired using an LTQ-Orbitrap Velos mass spectrometer controlled by Xcalibur 2.1 software (Thermo Fisher Scientific) and operated in data-dependent acquisition mode. The Orbitrap was set to analyze the survey scans at 60,000 resolution (at *m*/*z* 400) in the mass range *m*/*z* 300 to 2000, and the top 10 multiply charged ions in each duty cycle were selected for MS/MS in the LTQ linear ion trap. Charge state filtering, where unassigned precursor ions were not selected for fragmentation, and dynamic exclusion (repeat count, 1; repeat duration, 30 s; exclusion list size, 500) were used. Fragmentation conditions in the LTQ were as follows: normalized collision energy, 40%; activation *q*, 0.25; activation time 10 ms; and minimum ion selection intensity, 500 counts.

The raw data files were processed and quantified using Proteome Discoverer software version 1.4 (Thermo Fisher Scientific) and searched against the UniProt *P. falciparum* isolate 3D7 database (5365 entries) using the SEQUEST (Version 28 Revision 13) algorithm. Peptide precursor mass tolerance was set at 10 ppm, and MS/MS tolerance was set at 0.8 Da. Search criteria included carbamidomethylation of cysteine (+57.0214) as a fixed modification and oxidation of methionine (+15.9949) as a variable modification. Searches were performed with full tryptic digestion, and a maximum of 1 missed cleavage was allowed. The reverse database search option was enabled, and all peptide data were filtered to satisfy false discovery rate of 5%. Comparable eluates from a mirrored anti-FLAG resin using untagged parental 3D7 parasites were assayed as controls (see supplemental Dataset S1).

### Immunofluorescence assay of P. falciparum schizonts

Thin smears of *P. falciparum* schizont stage cultures were prepared on glass slides and allowed to air-dry. The smears were fixed in 4% paraformaldehyde, 0.01% glutaraldehyde/phosphate-buffered saline (PBS) for 1 h at room temperature. Fixed smears were then permeabilized with 0.1% Triton X-100 for 10 min at room temperature and blocked with 3% BSA/PBS for 1 h at room temperature. Staining was performed with primary rat anti-c-Myc (JAC6, Abcam) diluted 1:200 and primary rabbit anti-GAP45 (43) diluted 1:500, followed by secondary Alexa-Fluor-conjugated antibodies (Life Technologies, Inc., 1:500 dilution). All antibody dilutions were performed in 1% BSA/PBS. Confocal images were acquired using a Zeiss LSM 510 laser-scanning confocal microscope.

## Author contributions

C. S. B. cloned, expressed, and performed all *in vitro* characterizations of PfMyoA; C. L. T. and R. M. identified PfELC in parasites; M. J. P. performed mass spectrometry; E. B. K. performed analytical ultracentrifugation and protein expression; P. M. F. expressed and purified *Plasmodium* actin; H. L. assayed *Plasmodium* actin by TIRF microscopy; C. S. B., J. B., and K. M. T. designed the research and wrote the paper.

## Supplementary Material

Supplemental Data

## Footnotes

5 Please note that the JBC is not responsible for the long-term archiving and maintenance of this site or any other third party hosted site.

## Footnotes

4 The abbreviations used are:

MTIP: myosin tail-interacting protein
BisTris: 2-[bis(2-hydroxyethyl)amino]-2-(hydroxymethyl)propane-1,3-diol
PfELC: *P. falciparum* essential light chain
ACN: acetonitrile
AB: ammonium bicarbonate
MD: motor domain
TCEP: tris(2-carboxyethyl)phosphine
GAP: glideosome-associated protein.

## References

[1] World Health Organization (2016) 2014 World Malaria Report. World Health Organization, Geneva, Switzerland

[2] Tardieux I., and Baum J. (2016) Reassessing the mechanics of parasite motility and host-cell invasion. J. Cell Biol. 214, 507–515 27573462 10.1083/jcb.201605100 PMC5004448

[3] Bergman L. W., Kaiser K., Fujioka H., Coppens I., Daly T. M., Fox S., Matuschewski K., Nussenzweig V., and Kappe S. H. (2003) Myosin A tail domain interacting protein (MTIP) localizes to the inner-membrane complex of *Plasmodium* sporozoites. J. Cell Sci. 116, 39–49 12456714 10.1242/jcs.00194

[4] Bosch J., Turley S., Daly T. M., Bogh S. M., Villasmil M. L., Roach C., Zhou N., Morrisey J. M., Vaidya A. B., Bergman L. W., and Hol W. G. (2006) Structure of the MTIP-MyoA complex, a key component of the malaria parasite invasion motor. Proc. Natl. Acad. Sci. U.S.A. 103, 4852–4857 16547135 10.1073/pnas.0510907103 PMC1458759

[5] Bosch J., Turley S., Roach C. M., Daly T. M., Bergman L. W., and Hol W. G. (2007) The closed MTIP-myosin A-tail complex from the malaria parasite invasion machinery. J. Mol. Biol. 372, 77–88 17628590 10.1016/j.jmb.2007.06.016 PMC2702245

[6] Vahokoski J., Bhargav S. P., Desfosses A., Andreadaki M., Kumpula E. P., Martinez S. M., Ignatev A., Lepper S., Frischknecht F., Sidén-Kiamos I., Sachse C., and Kursula I. (2014) Structural differences explain diverse functions of *Plasmodium* actins. PLoS Pathog. 10, e1004091 24743229 10.1371/journal.ppat.1004091 PMC3990709

[7] Olshina M. A., Baumann H., Willison K. R., and Baum J. (2016) *Plasmodium* actin is incompletely folded by heterologous protein-folding machinery and likely requires the native *Plasmodium* chaperonin complex to enter a mature functional state. FASEB J. 30, 405–416 26443825 10.1096/fj.15-276618 PMC5423778

[8] Bookwalter C. S., Kelsen A., Leung J. M., Ward G. E., and Trybus K. M. (2014) A *Toxoplasma gondii* class XIV myosin, expressed in Sf9 cells with a parasite co-chaperone, requires two light chains for fast motility. J. Biol. Chem. 289, 30832–30841 25231988 10.1074/jbc.M114.572453 PMC4215259

[9] Hellerschmied D., and Clausen T. (2014) Myosin chaperones. Curr. Opin. Struct. Biol. 25, 9–15 24440450 10.1016/j.sbi.2013.11.002 PMC4045384

[10] Nebl T., Prieto J. H., Kapp E., Smith B. J., Williams M. J., Yates J. R. 3rd, Cowman A. F., and Tonkin C. J. (2011) Quantitative *in vivo* analyses reveal calcium-dependent phosphorylation sites and identifies a novel component of the *Toxoplasma* invasion motor complex. PLoS Pathog. 7, e1002222 21980283 10.1371/journal.ppat.1002222 PMC3182922

[11] Williams M. J., Alonso H., Enciso M., Egarter S., Sheiner L., Meissner M., Striepen B., Smith B. J., and Tonkin C. J. (2015) Two essential light chains regulate the MyoA lever arm to promote *Toxoplasma* gliding motility. MBio 6, e00845–15 26374117 10.1128/mBio.00845-15 PMC4600101

[12] Baca A. M., and Hol W. G. (2000) Overcoming codon bias: a method for high-level overexpression of *Plasmodium* and other AT-rich parasite genes in *Escherichia coli*. Int. J. Parasitol. 30, 113–118 10704592 10.1016/s0020-7519(00)00019-9

[13] Green J. L., Martin S. R., Fielden J., Ksagoni A., Grainger M., Yim Lim B. Y., Molloy J. E., and Holder A. A. (2006) The MTIP-myosin A complex in blood stage malaria parasites. J. Mol. Biol. 355, 933–941 16337961 10.1016/j.jmb.2005.11.027

[14] Bement W. M., and Mooseker M. S. (1995) TEDS rule: a molecular rationale for differential regulation of myosins by phosphorylation of the heavy chain head. Cell Motil. Cytoskeleton 31, 87–92 7553910 10.1002/cm.970310202

[15] Warshaw D. M. (2004) Lever arms and necks: a common mechanistic theme across the myosin superfamily. J. Muscle Res. Cell Motil. 25, 467–474 15630611 10.1007/s10974-004-1767-z

[16] Ghorbal M., Gorman M., Macpherson C. R., Martins R. M., Scherf A., and Lopez-Rubio J. J. (2014) Genome editing in the human malaria parasite *Plasmodium falciparum* using the CRISPR-Cas9 system. Nat. Biotechnol. 32, 819–821 24880488 10.1038/nbt.2925

[17] Warshaw D. M., Guilford W. H., Freyzon Y., Krementsova E., Palmiter K. A., Tyska M. J., Baker J. E., and Trybus K. M. (2000) The light chain binding domain of expressed smooth muscle heavy meromyosin acts as a mechanical lever. J. Biol. Chem. 275, 37167–37172 10945998 10.1074/jbc.M006438200

[18] Lowey S., Waller G. S., and Trybus K. M. (1993) Function of skeletal muscle myosin heavy and light chain isoforms by an *in vitro* motility assay. J. Biol. Chem. 268, 20414–20418 8376398

[19] Periz J., Whitelaw J., Harding C., Gras S., Del Rosario Minina M. I., Latorre-Barragan F., Lemgruber L., Reimer M. A., Insall R., Heaslip A., and Meissner M. (2017) *Toxoplasma gondii* F-actin forms an extensive filamentous network required for material exchange and parasite maturation. Elife 6, e24119 28322189 10.7554/eLife.24119 PMC5375643

[20] Koldewey P., Horowitz S., and Bardwell J. C. A. (2017) Chaperone-client interactions: Non-specificity engenders multifunctionality. J. Biol. Chem. 292, 12010–12017 28620048 10.1074/jbc.R117.796862 PMC5519353

[21] Frénal K., Jacot D., Hammoudi P. M., Graindorge A., Maco B., and Soldati-Favre D. (2017) Myosin-dependent cell-cell communication controls synchronicity of division in acute and chronic stages of *Toxoplasma gondii*. Nat. Commun. 8, 15710 28593938 10.1038/ncomms15710 PMC5477499

[22] Srikakulam R., and Winkelmann D. A. (2004) Chaperone-mediated folding and assembly of myosin in striated muscle. J. Cell Sci. 117, 641–652 14709723 10.1242/jcs.00899

[23] Bird J. E., Takagi Y., Billington N., Strub M. P., Sellers J. R., and Friedman T. B. (2014) Chaperone-enhanced purification of unconventional myosin 15, a molecular motor specialized for stereocilia protein trafficking. Proc. Natl. Acad. Sci. U.S.A. 111, 12390–12395 25114250 10.1073/pnas.1409459111 PMC4151768

[24] Wright M. H., Clough B., Rackham M. D., Rangachari K., Brannigan J. A., Grainger M., Moss D. K., Bottrill A. R., Heal W. P., Broncel M., Serwa R. A., Brady D., Mann D. J., Leatherbarrow R. J., Tewari R., et al (2014) Validation of *N*-myristoyltransferase as an antimalarial drug target using an integrated chemical biology approach. Nat. Chem. 6, 112–121 24451586 10.1038/nchem.1830 PMC4739506

[25] Rohrbach P., Friedrich O., Hentschel J., Plattner H., Fink R. H., and Lanzer M. (2005) Quantitative calcium measurements in subcellular compartments of *Plasmodium falciparum-*infected erythrocytes. J. Biol. Chem. 280, 27960–27969 15927958 10.1074/jbc.M500777200

[26] Tyska M. J., Dupuis D. E., Guilford W. H., Patlak J. B., Waller G. S., Trybus K. M., Warshaw D. M., and Lowey S. (1999) Two heads of myosin are better than one for generating force and motion. Proc. Natl. Acad. Sci. U.S.A. 96, 4402–4407 10200274 10.1073/pnas.96.8.4402 PMC16344

[27] Herm-Götz A., Weiss S., Stratmann R., Fujita-Becker S., Ruff C., Meyhöfer E., Soldati T., Manstein D. J., Geeves M. A., and Soldati D. (2002) *Toxoplasma gondii* myosin A and its light chain: a fast, single-headed, plus-end-directed motor. EMBO J. 21, 2149–2158 11980712 10.1093/emboj/21.9.2149 PMC125985

[28] De La Cruz E. M., and Ostap E. M. (2004) Relating biochemistry and function in the myosin superfamily. Curr. Opin. Cell Biol. 16, 61–67 15037306 10.1016/j.ceb.2003.11.011

[29] Behrmann E., Müller M., Penczek P. A., Mannherz H. G., Manstein D. J., and Raunser S. (2012) Structure of the rigor actin-tropomyosin-myosin complex. Cell 150, 327–338 22817895 10.1016/j.cell.2012.05.037 PMC4163373

[30] Dvorak J. A., Miller L. H., Whitehouse W. C., and Shiroishi T. (1975) Invasion of erythrocytes by malaria merozoites. Science 187, 748–750 803712 10.1126/science.803712

[31] Cronan J. E. Jr. (1990) Biotination of proteins *in vivo*. A post-translational modification to label, purify, and study proteins. J. Biol. Chem. 265, 10327–10333 2113052

[32] Li S. J., and Cronan J. E. Jr. (1993) Growth rate regulation of *Escherichia coli* acetyl coenzyme A carboxylase, which catalyzes the first committed step of lipid biosynthesis. J. Bacteriol. 175, 332–340 7678242 10.1128/jb.175.2.332-340.1993 PMC196146

[33] Lu H., Fagnant P. M., Bookwalter C. S., Joel P., and Trybus K. M. (2015) Vascular disease-causing mutation R258C in ACTA2 disrupts actin dynamics and interaction with myosin. Proc. Natl. Acad. Sci. U.S.A. 112, E4168–E4177 26153420 10.1073/pnas.1507587112 PMC4534267

[34] Philo J. S. (2000) A method for directly fitting the time derivative of sedimentation velocity data and an alternative algorithm for calculating sedimentation coefficient distribution functions. Anal. Biochem. 279, 151–163 10706784 10.1006/abio.2000.4480

[35] Kinose F., Wang S. X., Kidambi U. S., Moncman C. L., and Winkelmann D. A. (1996) Glycine 699 is pivotal for the motor activity of skeletal muscle myosin. J. Cell Biol. 134, 895–909 8769415 10.1083/jcb.134.4.895 PMC2120956

[36] Trybus K. M. (2000) Biochemical studies of myosin. Methods 22, 327–335 11133239 10.1006/meth.2000.1085

[37] Previs M. J., VanBuren P., Begin K. J., Vigoreaux J. O., LeWinter M. M., and Matthews D. E. (2008) Quantification of protein phosphorylation by liquid chromatography-mass spectrometry. Anal. Chem. 80, 5864–5872 18605695 10.1021/ac800337v PMC3050605

[38] Weith A., Sadayappan S., Gulick J., Previs M. J., Vanburen P., Robbins J., and Warshaw D. M. (2012) Unique single molecule binding of cardiac myosin binding protein-C to actin and phosphorylation-dependent inhibition of actomyosin motility requires 17 amino acids of the motif domain. J. Mol. Cell. Cardiol. 52, 219–227 21978630 10.1016/j.yjmcc.2011.09.019 PMC3246064

[39] Gibson D. G., Young L., Chuang R. Y., Venter J. C., Hutchison C. A. 3rd, Smith H. O. (2009) Enzymatic assembly of DNA molecules up to several hundred kilobases. Nat. Methods 6, 343–345 19363495 10.1038/nmeth.1318

[40] Trager W., and Jensen J. B. (1976) Human malaria parasites in continuous culture. Science 193, 673–675 781840 10.1126/science.781840

[41] Fidock D. A., and Wellems T. E. (1997) Transformation with human dihydrofolate reductase renders malaria parasites insensitive to WR99210 but does not affect the intrinsic activity of proguanil. Proc. Natl. Acad. Sci. U.S.A. 94, 10931–10936 9380737 10.1073/pnas.94.20.10931 PMC23535

[42] Lambros C., and Vanderberg J. P. (1979) Synchronization of *Plasmodium falciparum* erythrocytic stages in culture. J. Parasitol. 65, 418–420 383936

[43] Baum J., Richard D., Healer J., Rug M., Krnajski Z., Gilberger T. W., Green J. L., Holder A. A., and Cowman A. F. (2006) A conserved molecular motor drives cell invasion and gliding motility across malaria life cycle stages and other apicomplexan parasites. J. Biol. Chem. 281, 5197–5208 16321976 10.1074/jbc.M509807200

