# Supplementary material for: Reconstitution of the core of the malaria parasite glideosome with recombinant Plasmodium class XIV myosin A and Plasmodium actin
Source: J Biol Chem. 2017 Oct 4;292(47):19290–303. doi: 10.1074/jbc.M117.813972 (PMC5702669; doi:10.1074/jbc.M117.813972)
Supplement: Supplemental Data [file supp_292_47_19290__index.html]

Reconstitution of the core of the malaria parasite glideosome with recombinant Plasmodium class XIV myosin A and Plasmodium actin — Reconstitution of the core of the malaria parasite glideosome with recombinant Plasmodium class XIV myosin A and Plasmodium actin — Reconstitution of the malaria parasite glideosome — Supplemental Data 

# Reconstitution of the core of the malaria parasite glideosome with recombinant *Plasmodium* class XIV myosin A and *Plasmodium* actin

## Supplemental Data

- Supplemental information (.pdf, 47 KB) - Supplemental Figure 1, Supplemental Dataset 1 legend, and Supplemental Movie legends
- Supplemental Dataset 1 (.xlsx, 66 KB) - Supplemental Dataset 1
- Supplemental Movie 1 (.avi, 10.9 MB) - Supplemental Movie 1
- Supplemental Movie 2 (.avi, 10.9 MB) - Supplemental Movie 2
