# Supplementary material for: Reconstitution of the core of the malaria parasite glideosome with recombinant Plasmodium class XIV myosin A and Plasmodium actin
Source: J Biol Chem. 2017 Oct 4;292(47):19290–303. doi: 10.1074/jbc.M117.813972 (PMC5702669; doi:10.1074/jbc.M117.813972)
Supplement: Supplemental Data [file 10.1074_M117.813972_jbc.M117.813972-1.pdf]

Reconstitution of the core of the malaria parasite glideosome with recombinant *Plasmodium* class XIV myosin A and *Plasmodium* actin

Carol S. Bookwalter, Chwen L. Tay, Rama McCrorie, Michael J. Previs, Hailong Lu, Elena B. Krementsova, Patricia M. Fagnant, Jake Baum, and Kathleen M. Trybus

Includes: Supplemental Figure 1, Supplemental Dataset 1 legend, and Supplemental Movie Legends

```
MQEFVMNILSKEKIGKIEKLKNDGNELYRNKKYKEALCIYDNAVCEFCGGSGDSLKIKEMVKEF
RENGGKKEVDIPEGKSADSPHLNQHVKDLFIKICHNISLCYYFLEDFEKSIEYCLYINEMNNNH
YKSYHTLGLCYEKLKDYQKSIHYFDRCKIVLLKNQNNKNKDNNNKSEINRINEKLRDIMKIIDQ
NKNDPYKNISNIKKYLLDEASTEEEKIKLLHSIYNQKFYILLKENIFLFLFDFINHGENSLSLE
KTAIYVIYKILSKLDNEHIIIENSKDDNYNKNRNICINKLDNSKLQYYYDLDYIKMILSFNEYF
TKDWIYNYVKEKMKILENLKFSKDETLYKEHVDILIYIINIMKYVYVINNDYILNIINSYYLNS
DNSNINNSGINALTFLCKKKQFLTQNSKDNRGKNSITLLNTLLEDSNIDVKHYFIEFHFSDSHK
YPLCINSEIKKIIQNVIGMYEHFSSSIEYTLILIFTLLHDPQRPKEKDIEMNDVIYDCIDNYFH
HNENILIEWFVCIKCLFLVDKNIILNYLIGKTEYIVKILHFITNCIGRKTKEELSIYIDVLLLL
LNISEIRFMFTNYIDMYINIMKSLNYDQCFLKLLLGTFKLYMHNIDFKQQIQDNVDLFFYAKEI
LKQFLLRSEGQKNKISSDGGPTQKDSPTSLQKREFLESTEQSSHNGRKNCELVSKMMKKQTKIS
SNGNTDYDTHALKDLIEMLFYLSLHIEFKKQLLEEKNNYILFFLIKVGHDINKKKLDNTYKYIY
CNTINNLILTKNDEKIKRREINKTNLSNFDNEQIEALEQFYDKLPKEARPKTDPLYDYGDEETS
NKLIDLLLYNEKYVLKVKENADEEIILPKGGSYTNGTIINIIYNFINSNFFTTNIAESVCEIIS
KFVKNTNNIGIVLVNNGLKTLLLASKHITNKKNCALALSEIFIYTNPKLIHFYEAYDSLPLLIE
QLKSDEELLIFKTLMAITNILTIDENVAIKAMQLNLWYKCFDILSTENEYIKSASLECICNLCS
QSHVHQYIYDKYQTIMKSKNESDKDILFVDIQIIYSFTMEYQNYKCVFAATGALGMLSSDLRLP
YYLVRTKGIDHIFSSFNNTTDQNILLRILTFFNNIMTCDDIPDDILKKIKTYVEKKKDLNEENT
QMANFILQGSEQKLISEEDL
```

Supplemental Figure 1. Sequence of the chimeric PUNC chaperone used in this study. Sequences in black are from *P. falciparum* (accession number PF3D7_1420200, nomenclature according to http://PlasmoDB.org, or GenBank XP_001348369.1), and sequences in red are derived from *P. knowlesi* (PlasmoDB PKNH_1337800, or GenBank XP_002260808). The C-terminal Myc tag is shown in green.

Supplemental Dataset 1 Legend

Dataset S1. Supplementary excel spreadsheet for proteins identified by mass spectrometry from immunoprecipitation of PfMyoA-2cMyc-2FLAG lysate. Protein hits are listed from control 3D7 cultures (A) versus transgenic cultures in which PfMyoA is tagged with a 2cMyc-2FLAG C terminal tag. **Area** refers to the average area of the three unique peptides with the largest peak area. **Score** refers to the protein score, which is the sum of the scores of the individual peptides. **PSM** refers to the total number of identified peptide sequences (peptide spectrum matches) for the protein, including those redundantly identified. **AA**, amino acids of identified target. **MW**, predicted molecular weight. **Calculated pI** refers to the theoretically calculated isoelectric point. PfELC is highlighted in yellow.

Supplemental Movie Legends

Supplemental Movie 1

In vitro motility assay showing expressed *Plasmodium* actin filaments being moved by PfMyoA adhered to the coverslip. The filaments were stabilized by jasplakinolide and visualized with actin-chromobody-Emerald using TIRF microscopy. The bar is 3 µm long, and the movie is played at 4x real time. Conditions: Conditions: 25 mM imidazole, pH 7.5, 125 mM KCl, 1 mM EGTA, 4 mM $MgCl_2$, 10 mM DTT, 2 mM MgATP, 30°C.

Supplemental Movie 2

In vitro motility assay showing skeletal muscle actin filaments being moved by PfMyoA adhered to the coverslip. The filaments were stabilized by jasplakinolide and visualized with actin-chromobody-Emerald using TIRF microscopy. The bar is 3 µm long, and the movie is played at 4x real time. Conditions: Conditions: 25 mM imidazole, pH 7.5, 125 mM KCl, 1 mM EGTA, 4 mM $MgCl_2$, 10 mM DTT, 2 mM MgATP, 30°C.
